# Student productivity log: An innovative approach to managing clinic sessions with no patient

**DOI:** 10.1002/jdd.13596

**Published:** 2024-05-25

**Authors:** Tara L. Newcomb, Jessica Suedbeck, Lauren Eusner, Amber W. Hunt

**Affiliations:** ^1^ Gene W. Hirschfeld School of Dental Hygiene Old Dominion University Norfolk Virginia USA

**Keywords:** allied dental education, clinical dental hygiene education, practice managment

## 1 PURPOSE

**FIGURE 1:**
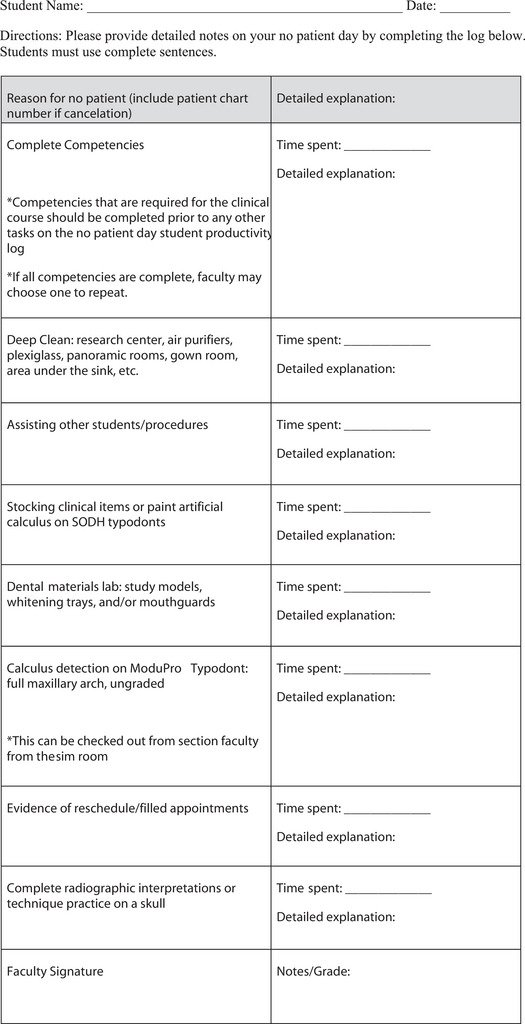
Student productivity log.

Every effort must be made by students to complete tasks in clinical courses that support competency requirements and participation.^1^Participation is essential for every session of clinical courses, regardless of patient presence. The purpose of the Student productivity log (SPL) was to better manage and document student progress during clinic sessions with no patient due to cancellations or inability to fill the appointment (Figure 1).

## 2 METHODS

Senior dental hygiene students enrolled in a clinical course were required to complete an SPL on no‐patient days. The SPL prompted students to document the completion of four tasks and/or four clinical competencies, or some combination for a minimum of 2.5‐h of work during their 3‐h clinic session. SPL collected information on the reason for no patient during the session and time used to complete each task. The SPL included required tasks of 1) completing non‐patient clinical competencies, 2) performing practice management tasks (similar to tasks completed with patient cancellations in private practice)^2^ ^,^ ^3^, and 3) simulated practice of clinical skills. Section faculty advised students on a plan for completing the SPL and students were required to complete tasks and competencies in their assigned operatory (unless designated to another location, such as the dental materials lab). Students who did not meet minimum criteria for completing the SPL would receive a one‐point deduction from their final grade in the course. Students were required to complete all parts of the log using complete sentences. Faculty were required to sign the SPL and turn into clinic course director.

## 3 OUTCOMES

By mid‐semester, a total of 77 logs were turned in by a class of 37 senior dental hygiene students completing 79 competencies and 14 simulated practice skills–more specifically, 1 student completed radiographic technique practice on a skull and 13 completed calculus detection exercises using ModuPro typodont. The most completed tasks were competencies, scheduling patients, and use of the materials lab. The last completed tasks were practicing the radiographic technique on a skull, calculus detection exercises, and deep cleaning of any space in the clinic. Directions for faculty were mostly followed with only nine faculty not signing the document. Directions on writing complete sentences and listing reasons for cancellations were directions most often not followed by students. Only 1 student received −1 point off their grade for not completing all requirements for the SPL.

## 4 CONCLUSIONS/SIGNIFICANCE

The SPL was utilized by students and faculty during the Spring 2024 semester. A diverse number of clinical competencies, practice management tasks, and skill practice exercises were completed and well‐documented by faculty and students. Clinic course directors and advisors were able to review logs to advise students on the use of clinic time. In addition, the SPL gave faculty a way to assess participation and guided students toward using no‐patient clinic sessions in a way that optimized their course participation.
